# Targeting the redox-programmed cell death axis in breast cancer: from molecular mechanisms to therapeutic resistance

**DOI:** 10.1038/s41420-025-02743-y

**Published:** 2025-10-06

**Authors:** Yiqiao Wen, Zhixuan Lin, Zhongwei Jiang, Yang Li, Tianyi Wu

**Affiliations:** 1 The Forth Clinical College of China Medical University, Shenyang, China; 2 Department of Urology, The Third Affiliated Hospital of Shenyang Medical College (Shenyang 242 Hospital), Shenyang, China; 3 Department of Gynecology, Shengjing Hospital of China Medical University, Shenyang, China; 4 Department of Pathophysiology, College of Basic Medical Sciences, China Medical University, Shenyang, China

**Keywords:** Cell death, Breast cancer

## Abstract

Breast cancer, the most prevalent malignancy among females, threatens public health worldwide. Patients with breast cancer need personalised treatment strategies on the basis of their distinct molecular characteristics due to the unique epidemiological patterns and high heterogeneity of breast cancer, which limits therapeutic efficacy and poses significant challenges to current treatments. The underlying reasons may involve complex interactions and alterations in various cell death pathways. Currently, most studies and therapeutic agents focus on a single type of cell death, whereas opportunities related to other cell death pathways are typically overlooked. Therefore, identifying the predominant type of cell death, understanding the transitions between different cell death modalities during treatment, and developing novel therapies are crucial. In this review, we summarise the dynamic balance between reactive oxygen species (ROS) production and clearance, as well as the characteristics of various forms of cell death induced by ROS, including pyroptosis, apoptosis, necroptosis, autophagy, ferroptosis, cuproptosis, disulfidoptosis, oxeiptosis, and epigenetic regulation of these types of cell death. Additionally, we explored a novel cell death pathway called PANoptosis. This review sheds new light on the treatment of breast cancer from the perspective of nanotechnology and the development of combination therapies.

## Facts

- Introducing PANoptosis and constructing a comprehensive network of cell death mechanisms
- The key role of ROS metabolic imbalance in the occurrence, development and treatment resistance of breast cancer
- Taking ROS as the core node connecting cell death pathways and therapeutic strategies
- Exploring innovative therapeutic strategies targeting the ROS-cell death network, providing a new direction for the precise treatment of breast cancer

## Open questions

- Can we design ‘epigenetic triggers’ to selectively prime resistant cells for programmed cell death execution?
- How to precise molecular thresholds determining the shift from pro-survival signalling to lethal programmed cell death execution?
- How can we engineer ‘ programmed cell death -cascade nanoparticles’ that leverage tumour microenvironment signals?

## Introduction

Despite the overall decline in cancer mortality rates, the annual morbidity of breast cancer is increasing, and breast cancer is currently the most prevalent cancer globally and the second leading cause of cancer-related deaths among females [1]. A variety of treatment modalities are available for breast cancer, including surgical intervention, antiestrogen therapy for hormone receptor (HR)-positive cases, chemotherapy, targeted therapy, and the integration of immunotherapy [2]. Given its high degree of heterogeneity, treatment strategies must be tailored to specific molecular characteristics [3]. Recent research has identified oxidative hotspots of hydrogen, laying a foundation for understanding cancer cell migration [4]. Reactive oxygen species have been determined to be critical inducers of various forms of cell death, which are collectively termed oxidative cell death [5]. Given its intimate association with ROS, cell death can be triggered by genetically programmed mechanisms (such as apoptosis, necrosis, and pyroptosis), metabolic dysfunction, including ferroptosis, cuproptosis, oxytosis, disulfidptosis [6] and the recently identified sodium-dependent cell death [7]. Strategies that target ROS and utilise nanoparticles to increase cell death have emerged as innovative strategies for cancer treatment [8].

The initiation, progression, and drug resistance mechanisms of tumours are dynamically regulated by multiple programmed cell death (PCD) pathways [9]. Epigenetic regulatory mechanisms, as pivotal molecular switches, mediate numerous biological processes, including cell death [9]. This study delves into the mechanisms underlying breast cancer and ROS-related cell death, along with the associated epigenetic regulatory network, and systematically analyses the impact of different PCD modalities on tumour evolution and treatment, shedding new light on the optimisation of clinical treatment strategies and the development of emerging nanoparticle-targeted cell death therapies.

## ROS

ROS are signalling molecules composed of oxygen-containing reactive entities such as the superoxide anion (O_2_^−^), singlet oxygen (^1^O_2_), the hydroxyl radical (·OH) and hydrogen peroxide (H_2_O_2_), and primarily originate from electron leakage in the mitochondrial electron transport chain [10].

### Mechanisms of ROS production

In addition to mitochondrial sources, ROS generation involves various enzymatic pathways: transmembrane electron transfer mediated by NADPH oxidase (NOX), substrate oxidation catalysed by xanthine oxidoreductase (XOR), and byproducts from lipoxygenase (LOXs) and cyclooxygenase (COX) during arachidonic acid metabolism [11].

NOX4, a key source of ROS, serves as the predominant NADPH oxidase enzyme in breast cancer and facilitates oxidative stress regulation [12]. A distinctive feature of NOX4 lies in its ability to generate ROS within the inner membrane via the p22phox protein without activating cytoplasmic oxidase proteins or GTPase Rac [13], thereby promoting the metastasis of breast cancer through lymphangiogenesis [12]. Peroxisomes contain key enzymes such as catalase and flavoprotein oxidase, which are involved in fatty acid α-oxidation, fatty acid β-oxidation, and purine metabolism, as well as the biosynthesis of glycerides and bile acids. In addition to its role in purine catabolism, XOR exhibits nitrite reductase activity to produce nitric oxide and NADH oxidase activity to generate ROS under hypoxic, acidic, and inflammatory conditions [14]. The metabolites produced by arachidonic acid (AA) via the LOX and COX pathways can induce the production of ROS by activating NOX [14, 15]. Specifically, bioactive molecules such as leukotrienes and prostaglandins produced during AA metabolism via LOX and COX can directly or indirectly increase NOX complex activity, leading to the catalysis of intracellular ROS synthesis, including O₂^−^, thereby participating in the regulation of oxidative stress and cell signalling pathways [15]. Notably, ionising radiation significantly elevates intracellular endogenous ROS levels through DNA damage in a dose-dependent manner [16].

### Elimination of ROS

**Fig. 1:**
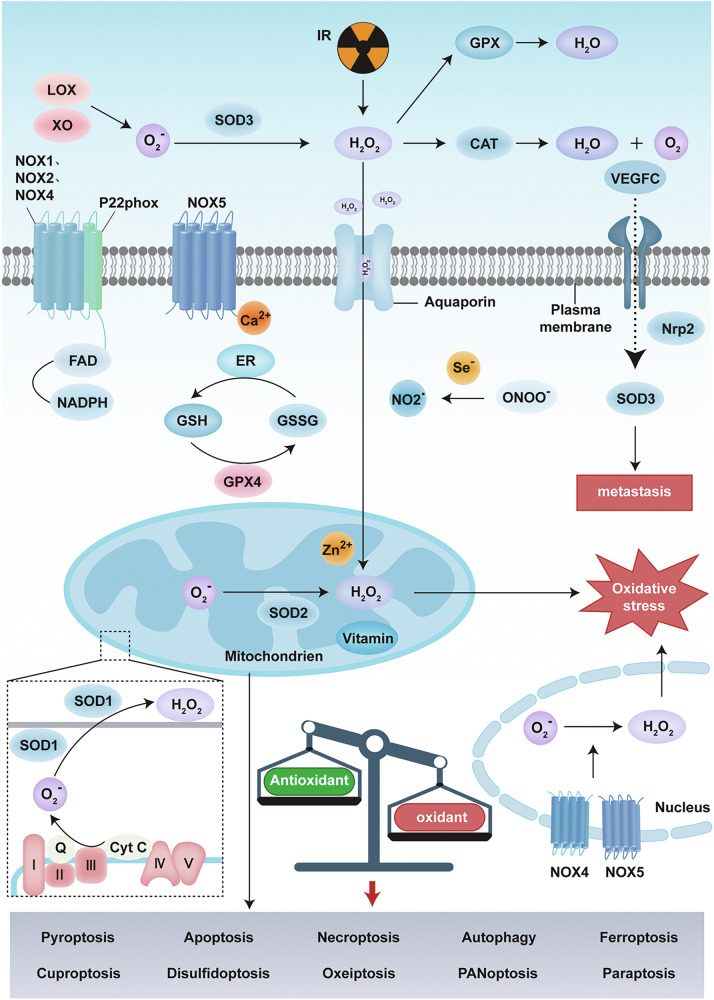
Intracellular signalling pathways related to oxidative stress and various forms of cell death triggered thereby. NOX1, NOX2, NOX4, NOX5 and other enzymes are located on the cell membrane and catalyse the production of superoxide anions, with FAD and NADPH acting as cofactors. P22phox is a component of the NOX complex. Peroxidase 4 (GPX4) is involved in this process. The endoplasmic reticulum (ER), selenium (Se), Zn^2+^ and vitamins are involved in regulating the process of antioxidation. In mitochondria, SOD2 converts superoxide anions to hydrogen peroxide. If the balance between oxidation and antioxidation is disrupted, oxidative stress can be triggered. O₂^−^: Superoxide anions; O₂: Oxygen; H₂O₂: Hydrogen peroxide; ONOO^−^: Peroxynitrite; NO₂^−^: Nitrite; SOD: Superoxide Dismutase; LOX: Lipoxygenase; NOX: NADPH oxidase; cytC: Cytochrome C; IR: Ionising radiation. The figure was created with BioRender.com.

Excessive ROS production can induce oxidative stress despite its crucial physiological roles in the immune system and in cell signalling. Organisms have evolved a sophisticated antioxidant defence mechanism to counteract the detrimental effects of ROS [17]. This multitiered defence system comprises an endogenous antioxidant enzyme system (e.g., SOD/CAT/GPx) and small-molecule free radical scavengers such as vitamin C/E and β-carotene, which are supported by trace elements such as selenium and zinc, collectively maintaining redox homeostasis [18]. Therefore, in Fig. 1, we summarise the production and regulation of ROS, including both intracellular and extracellular ROS generation, as well as the associated regulatory systems.

#### Superoxide dismutase (SOD)

SOD is an integral part of the endogenous antioxidant defence system in organisms and figures prominently in regulating ROS homeostasis by catalysing the disproportionation of O₂^−^ into O₂ and H₂O₂ [19]. SOD reduces intracellular superoxide anion levels, thereby mitigating ROS-induced damage [20]. Specifically, studies have shown that SOD1 is abnormally overexpressed in breast cancer and is significantly correlated with ErbB2 oncogene activation and elevated ROS levels [21]. Notably, despite the upregulation of SOD1 in the high-ROS subgroup of ErbB2-positive breast cancer cells, normal cellular proliferation still relies on SOD1 activity, indicating its dual role in promoting cancer progression and maintaining basal metabolism [22]. Furthermore, the function of SOD2 is concentration dependent: when the intracellular O₂^−^ level exceeds a specific threshold, the activity of SOD2 may exacerbate oxidative damage [22]. Therefore, regulating tumour progression hinges on maintaining ROS below the critical threshold that supports oncogene dependence [23], on the basis of which targeted regulation of SOD activity is regarded as a potential strategy in cancer treatment [24]. SOD-3 is a downstream effector of VEGFC, and VEGFC mediates SOD-3 through Nrp2 [24, 25]. SOD-3 and VEGFC are involved in the metastasis of breast cancer, and their expression is positively correlated with cancer [25].

#### Catalase (CAT)

CAT, an enzyme that decomposes H_2_O_2_ into O_2_ and H_2_O, is involved in the antioxidant defence mechanism [25]. In MCF-7 cancer cells, the overexpression of CAT affects their proliferation and migration and reduces their sensitivity to anticancer treatment [25]. Research has shown that CAT is associated with the luminal B subtype of breast cancer [26]. Reduced CAT activity has been observed in breast cancer; however, its activity is positively correlated with the advanced invasion and metastasis phenotypes of breast cancer in vivo [18].

#### Glutathione peroxidase (GPX)

The GPX family comprises eight members (GPX1–GPX8), which serve as classic antioxidant enzymes to mitigate oxidative stress and maintain redox homeostasis [27]. GPX specifically catalyses the reduction of lipid hydroperoxides (LOOHs) and organic hydroperoxides (ROOHs) through glutathione (GSH)-dependent reactions, thereby minimising oxidative damage and regulating prostaglandin biosynthesis [28]. Selenoproteins can catalyse the decomposition of peroxynitrite (ONOO^−^) into nitrite (NO₂^−^) and significantly reduce ONOO^−^-mediated protein nitration and DNA damage, thereby increasing cellular resistance to nitrogen stress [29].

#### Glutathione reductase (GR)

GR plays a crucial role in maintaining optimal glutathione levels by catalysing the reduction of oxidised glutathione (GSSG) to its reduced form, GSH, which is the primary antioxidant within the cellular antioxidant system [29], thereby preserving cellular redox homeostasis [30]. Studies have demonstrated that GR is highly expressed in MCF-7 breast cancer cells and that elevated GR activity is associated with increased resistance to radiotherapy; thus, inhibiting GR sensitises cells to oxidative stress [31].

#### Small-molecule free radical scavengers and trace elements

Vitamin D exerts its antioxidant effects by counteracting the activity of NADPH oxidase, which is responsible for ROS production [32]. Vitamin D also enhances the overall antioxidant capacity by upregulating the activity of peroxidases such as superoxide dismutase [32, 33]. Zn^2+^ regulates oxidative stress by inhibiting NOX [34]. Studies have revealed that the accumulation of zinc can increase the production of mitochondrial ROS, which in turn activates NF-κB and consequently modulates NOX1 expression [35].

## Breast cancer

The molecular classification of breast cancer is determined by the status of hormone receptors. The prognostic outcomes for each subtype exhibit substantial variability [36], making it imperative to establish differentiated follow-up monitoring strategies on the basis of molecular typing, particularly for HER2-positive and triple-negative subtypes, which require enhanced posttreatment imaging surveillance and circulating tumour DNA detection. Future research should concentrate on analysing intrasubtype molecular heterogeneity and developing novel targeted therapies [37], especially therapeutic breakthroughs in triple-negative breast cancer [38]. Table 1 summarises the regulatory mechanisms of noncoding RNAs in the PCD of breast cancer.

**Table 1:** Regulatory mechanisms of noncoding RNAs in PCD of breast cancer.

ncRNAs	Expression	Mechanism	Role in PCD	Reference(s)
circ_0022587	Down	Target miR-335-5p /Phosphoglycerate Kinase 1 Pathway	Promote apoptosis	[162]
LURAP1L-AS1	Up	Modulate oncogenes including EZH2, MCL1, and KRAS,	Promote apoptosis	[163]
miR-223-3p	Up	Target and downregulate *KIF4A*	Promote apoptosis	[164]
lncRNA AC112721.1	Down	Bind to THBS1 and regulate miR-491-5p/C2CD2L axis	Inhibit apoptosis	[165]
hsa-miR-214-3p	Up	Downregulate B7H3	Promote apoptosis	[166]
miR-484	Up	Suppress apoptosis in BC cells	Inhibit apoptosis	[167]
miR-454-3p	Up	Target ACSL4	Inhibit ferroptosis	[168]
miR-128-3p	Up	Suppress SP1 and CD98hc	Promote ferroptosis	[169]
lncRNA PTPRG-AS1	Up	Inhibit ferroptosis in breast cancer via miR-376c-3p/SLC7A11 axis	Inhibit ferroptosis	[170]
circ_0000643	Up	Enhance the expression of SLC7A11	Inhibit ferroptosis	[171]
LINC00460	Up	Regulate LINC00460/miR-320a/MAL2 axis	Inhibit ferroptosis	[172]
LINC01614	Up	Upregulate SLC31A1	Promote cuproptosis	[103]
miR-335	Down	SOD2 overexpression	Promote pyroptosis	[173]
Circ_0022382	Up	Activate PI3K/AKT/mTOR signalling pathway and SLC7A11	Promote disulfidptosis	[174]
miR-141-3p	Up	Target RaAB10	Inhibit autophagy	[175]
miR-216b	Down	Target HK2	Promote autophagy	[175]

## Molecular mechanisms of different cell death pathways

### PANoptosis

**Fig. 2:**
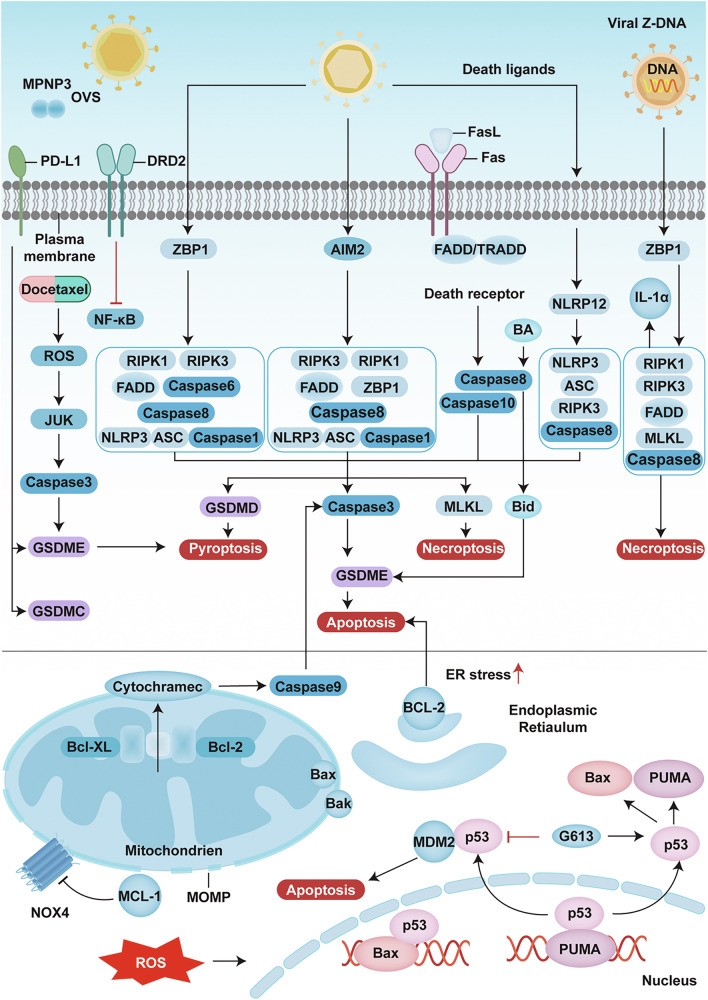
PANoptosis signalling pathways triggered by various stimuli in cells. The PANoptosome can concurrently participate in three key programmed cell death modes: apoptosis, pyroptosis and necroptosis. When triggered by certain factors, sensors, including ZBP1, AIM2, RIPK1 and NLRP12, can interact and recruit several other molecules to form PANoptosomes, which then induce the progression of PANoptosis. AIM2: absent in melanoma 2; ASC: Apoptosis-associated speck-like protein containing a caspase recruitment domain; CASP: Caspase; FADD: Fas-associated death domain protein; GSDMD: Gasdermin D; GSDME: Gasdermin E; GSDMC: Gasdermin C; MLKL: Mixed lineage kinase domain-like pseudokinase; RIPK: Receptor-interacting serine/threonine-protein kinase; ZBP1: Z-DNA binding protein 1; MOMP: Membrane permeabilisation. The figure was created with BioRender.com.

PANoptosis represents an integrated form of cell death encompassing apoptosis, pyroptosis, and necrosis [39], which is orchestrated by the PANoptosome complex. To date, four primary types of PANoptosomes have been identified: the ZBP1-PANoptosome (comprising ZBP1, NLRP3, ASC, caspase-1, caspase-6, caspase-8, RIPK1 and RIPK3) [39], AIM2-PANoptosome (comprising AIM2, Pyrin, ZBP1, ASC, caspase-1, caspase-8, FADD, RIPK1 and RIPK3) [40], RIPK1-PANoptosome (comprising RIPK1, RIPK3, NLRP3, ASC, caspase-1 and caspase-8) [41] and NLRP12-PANoptosome (comprising NLRP12, ASC, caspase-8 and RIPK3) [42]. These complexes facilitate the activation of caspase-3/7, cleavage of GSDMD and GSDME, and phosphorylation of MLKL, leading to membrane pore formation and the progression of PANoptosis [39]. Given the high heterogeneity of breast cancer, continuous refinement of existing molecular typing systems and prognostic assessment tools is needed. A robust prognostic prediction model, validated across multiple dimensions, has been developed [43]. This model can effectively guide the prediction of chemotherapy sensitivity and optimisation of targeted-immune combination therapies, thereby providing molecular tools to advance precise medicine paradigms in breast cancer management [44]. PANoptosis cannot be suppressed by pyroptosis, apoptosis or necroptosis. The formation and activation of the PANoptosome within a single cell also support PANoptosis. The molecular and regulatory mechanisms of this process are shown in Fig. 2.

#### Apoptosis

As the central regulatory hub of apoptosis, mitochondria mediate signal transduction through both intrinsic and extrinsic pathways. The extrinsic pathway is initiated by death receptor-mediated or granzyme-dependent mechanisms [45]. Specifically, the extrinsic pathway begins with the binding of death receptors, members of the TNFR superfamily such as Fas and DR4/5, to their respective ligands, such as FasL and TRAIL. This binding subsequently recruits and activates initiator caspases-8/10 via adaptor proteins such as FADD/TRADD, leading to downstream activation of the effector caspases-3/7 and culminating in apoptosis [43]. The intrinsic pathway involves mitochondria-related and endoplasmic reticulum stress pathways, where the Bcl-2 protein family plays a pivotal role by modulating mitochondrial outer membrane permeabilisation (MOMP) [46]. This family includes antiapoptotic members (e.g., Bcl-2 and Bcl-xL) and proapoptotic members (e.g., Bax and Bak). Upon activation, proapoptotic proteins form pore complexes on the mitochondrial membrane, resulting in the loss of the mitochondrial membrane potential and the release of apoptotic factors such as cytochrome C, thereby activating the caspase-9/3 cascade [46]. Dysregulated apoptosis signalling pathways in breast cancer cells can be reprogrammed to re-enter the apoptotic cycle, representing a critical therapeutic strategy. In breast cancer, overexpression of the antiapoptotic protein MCL1 impedes apoptosis by inhibiting mitochondrial NOX4 function. However, BH3 mimetics targeting MCL1 have demonstrated promising therapeutic potential [47]. Moreover, p53, a key tumour suppressor, exerts dual regulatory effects on ROS-induced apoptosis. Wild-type p53 promotes apoptosis through the transcriptional activation of proapoptotic genes such as Bax and PUMA [48]. In triple-negative breast cancer (TNBC), mutant p53 induces nonclassical apoptosis via aberrant interaction with MDM2, independent of wild-type p53 [49]. Compound G613 promotes the apoptosis of MCF-7 cells by inhibiting the formation of the p53-MDM2 complex and upregulating p53 expression [50]. Additionally, increasing the bioavailability of bile acid promotes the apoptosis of breast cancer cells by activating the caspase-8/Bid/ROS pathway, suggesting a novel therapeutic target for hormone receptor-negative breast cancer [51].

Apoptotic pathways interactively regulate other cell death modalities. In ferroptosis–apoptosis cross-regulation, long noncoding RNA LINC00618 regulates cell death through a dual mechanism: downregulating SLC7A11 to inhibit ferroptosis and upregulating BAX expression to promote apoptosis [52]. In autophagy-dependent apoptosis, high ROS levels in tumour cells enhance the sensitivity to apoptosis by inducing mitochondrial hyperpolarisation, curcumin or DNA damage [53].

#### Necroptosis

Necroptosis is a caspase-independent form of cell death that significantly regulates the initiation and progression of breast cancer [54]. Necroptosis is a precisely regulated form of programmed necrosis, and its core mechanism hinges on the assembly of the RIPK1/RIPK3 kinase complex and subsequent phosphorylation–oligomerisation of MLKL. In triple-negative breast cancer, RIPK1 facilitates vascular mimicry by activating the AKT/eIF4E signalling pathway [55]. Moreover, RIPK3 expression exhibits spatiotemporal heterogeneity [56]. Owing to hypermethylation of the promoter, RIPK3 is silenced in primary tumours but significantly upregulated in recurrent lesions, with an enhanced dependence on cysteine metabolism, suggesting that targeting RIPK3 may inhibit tumour recurrence [56].

Notably, the DNA damage response protein MRE11 activates the cGAS‒STING pathway, thereby driving necroptosis mediated by the ZBP1‒RIPK3‒MLKL axis [57]. Low ZBP1 expression in TNBC is significantly associated with genomic instability, an immunosuppressive microenvironment, and a dismal prognosis, indicating its potential as a biomarker for functional defects in this pathway [58]. Necroptosis plays a dual role in tumour immune regulation, as it can activate antigen-specific immune responses by releasing damage-associated molecular patterns, while RIPK3/MLKL-mediated IL-1α release can suppress T-cell function and promote tumour immune escape [59]. In light of this dichotomy, novel targeted nanocomplexes have been developed to induce immunogenic cell death by specifically increasing RIPK3 phosphorylation and MLKL tetramerisation, thereby enhancing the cytotoxic effect of T cells on triple-negative breast cancer [60]. This paradoxical immune regulation underscores the need for precise modulation of necroptosis according to the treatment stage: leveraging its immune-stimulatory properties in the early stages while inhibiting its metastasis-promoting effects in the later stages [61]. For example, lung metastasis can be significantly reduced by inhibiting MLKL [62]. Epigenetic regulation also plays a crucial role in necroptosis. Z-DNA binding protein 1 has emerged as a potential therapeutic target for modulating the necrosis process in advanced tumours [63]. Moreover, necroptosis-related lncRNAs, such as LINC00472, which regulates RIPK1, have demonstrated their predictive value for the prognosis of patients with breast cancer and their guiding value for personalised treatment strategies [63].

#### Pyroptosis

Pyroptosis is a form of PCD, and its classical activation pathway is mediated by caspase-1 and executed through the formation of membrane pores by proteins from the gasdermin (GSDM) family [64]. The canonical pyroptosis pathway is initiated by the activation of the NLRP3 inflammasome complex, which includes NLRP3, the adaptor protein ASC containing the CARD domain, and pro-caspase-1 [65]. Notably, inflammatory responses play a dual role in tumorigenesis: they can both promote malignant transformation and metastasis and exert an antitumour effect via pyroptosis induction [66]. The nonclassical pyroptosis pathway is mediated by caspase-4/5/11. Although these caspases cannot directly cleave the precursors of IL-1β/IL-18, they can still trigger pyroptosis by cleaving GSDMD-NT, a process specifically activated by bacterial lipopolysaccharides [67, 68]. Additionally, caspase-8 regulates pyroptosis under specific conditions: nucleus-localised PD-L1 induces pyroptosis via caspase-8-mediated cleavage of GSDMC in the hypoxic tumour microenvironment [69]. Docetaxel cleaves GSDME via the ROS/JNK/caspase-3 pathway, and its pyroptotic effect can be enhanced by demethylation of the DFNA5 gene, highlighting the role of epigenetic regulation in chemosensitisation [70]. In the context of triple-negative immunotherapy for breast cancer, the combination of pyroptosis induction with immune checkpoint inhibitors has significant synergistic potential [62]. For example, TAT3 inhibitor nanoparticles (MPNPs) in conjunction with *oncolytic viruses* can activate GSDME-dependent pyroptosis and markedly enhance the efficacy of anti-PD-1 therapy [62]. The dopamine receptor DRD2 has been identified as a novel therapeutic target to remodel the immunosuppressive tumour microenvironment by promoting M1 macrophage polarisation, inhibiting the NF-κB signalling pathway, and inducing pyroptosis in breast cancer cells [62], which provides a promising new treatment direction for breast cancer. These findings establish a robust theoretical foundation and translational strategy for precision medicine approaches targeting pyroptosis regulation in breast cancer. Epigenetic modifications also play crucial roles in mediating tumour growth and metastasis by regulating pyroptosis-related pathways [71].

### Ferroptosis

**Fig. 3:**
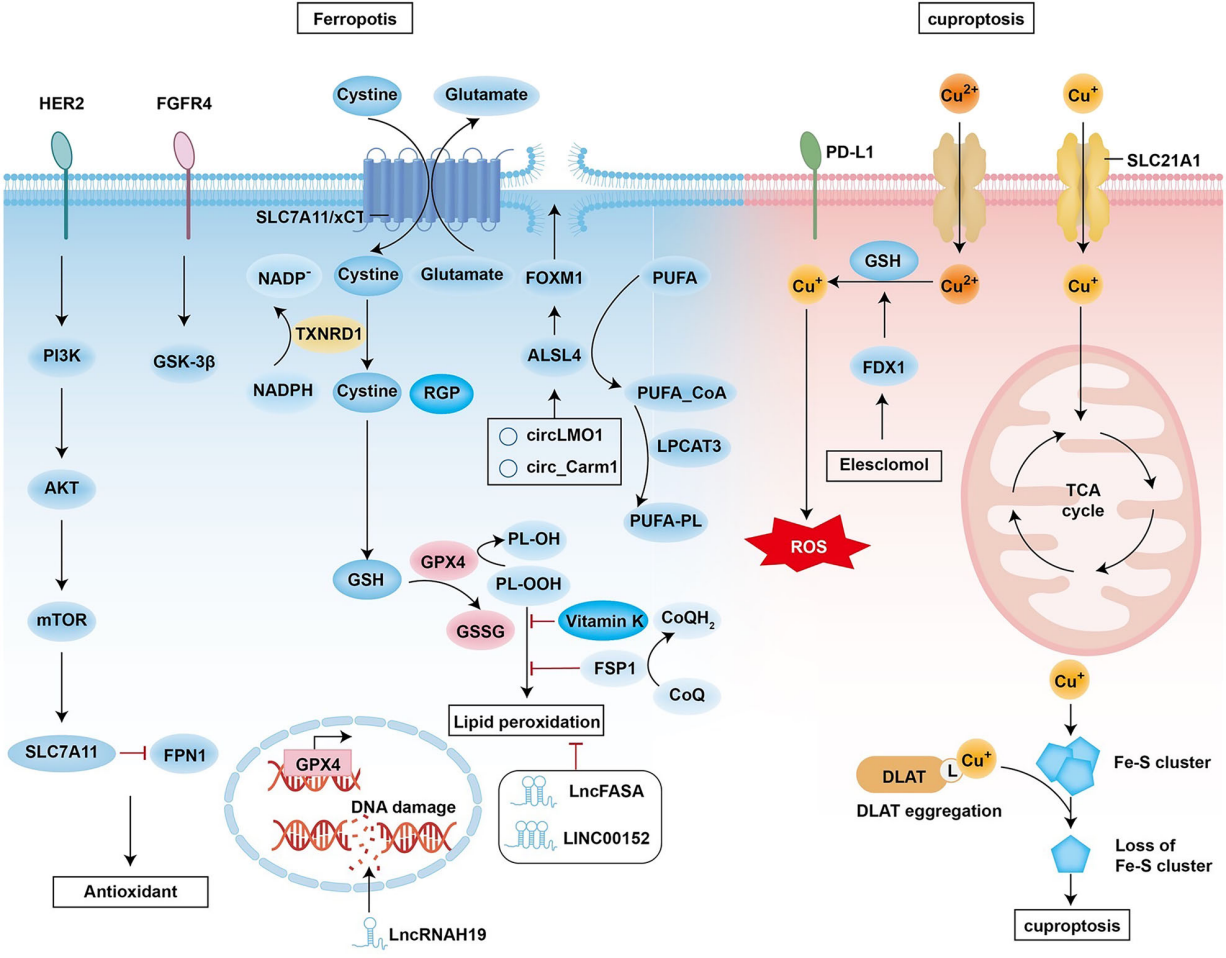
The mechanism of ferroptosis in cells and the related metabolic and signal transduction pathways. The synthesis of PUFA-PL, iron metabolism and mitochondrial metabolism can cause lipid peroxidation and induce ferroptosis. Conversely, the GPX4-GSH, FSP1-CoQH and DHODH-CoQH2 systems can inhibit lipid formation and thereby suppress ferroptosis. Imbalance of copper homeostasis can lead to an increase in intracellular Cu^+^ concentration and alter a series of cellular signalling pathways. Additionally, noncoding RNA is involved in copper metabolism. FSP1: Ferroptosis suppressor; GSSG: Glutathione; GSR: Glutathione-disulfide reductase; PUFA: Polyunsaturated fatty acid; PL-OOH: Phospholipid hydroperoxide; ROS: Reactive oxygen species; TCA: Mitochondrial TCA cycle. The figure was created with BioRender.com.

Ferroptosis is a form of iron-dependent PCD characterised by the aberrant accumulation of lipid peroxides and specific adjustments, as shown in Fig. 3 [72]. This process is specifically triggered by the peroxidation of polyunsaturated fatty acids (PUFAs), rather than a generalised increase in ROS [72]. Its regulatory network includes three core axes: the Xc^−^/GSH/GPX4 antioxidant system, ACSL4/LPCAT3/15-LOX pathway, and FSP1/CoQ10/NAD(P)H axis, which counteract ferroptosis by inhibiting lipid peroxidation [73]. Moreover, pathways mediating ferroptosis in breast cancer include T-cell inhibition via GCH1-BH4 [74] and ferroptosis triggered by DHODH-CoQH2 following GPX4 inactivation [75]. To date, 8 glutathione peroxidases have been identified, among which GPX4 directly reduces lipid peroxides [27], making it a potential therapeutic target for breast, ovarian, liver, and prostate cancers [76]. In breast cancer, the Xc^−^/GSH/GPX4 system is essential for maintaining redox homeostasis [77]. Cystine enters cells through Xc^−^ and is reduced to cysteine via the cystine reduction pathway dependent on GSH or thioredoxin reductase 1 (TXNRD1), thereby promoting GSH synthesis [78]. GSH, as a cofactor of GPX4, facilitates the reduction of phospholipid hydroperoxides (PLOOHs) to their corresponding alcohols (PLOHs), thereby maintaining redox balance [33].

Targeted inhibition of the Xc^−^/GSH/GPX4 system axis effectively induces ferroptosis [79]. For example, DMOCPTL regulates EGR1 in TNBC cells and induces GPX4 ubiquitination to reduce EGR4 protein levels, thereby regulating mitochondria-mediated apoptosis [80]. In the FSP1/CoQ10/NAD(P)H axis, FSP1 reduces CoQ10 to generate the antioxidant CoQH2 independently of GPX4, inhibits lipid peroxidation [81], and cooperates with vitamin K metabolism to protect cells [82]. Ferroptosis in breast cancer involves subtype-specific regulation. In triple-negative breast cancer, the expression level of GPX4 is significantly associated with prognosis. Studies have demonstrated that GPX4 is markedly upregulated in the luminal androgen receptor subtype of TNBC, thereby conferring resistance to ferroptosis [83]. Conversely, the basal-like subtype is more susceptible to ferroptosis induction due to elevated expression levels of ACSL4 and FADS1/2 [84]. Notably, the sensitivity of BRCA1-deficient breast cancer cells to PARP inhibitors can be increased by inhibiting GPX4, thereby providing a novel strategy to overcome drug resistance [75].

In luminal breast cancer, ER/PR positivity is positively correlated with GPX4 expression, while m6A demethylation activates SLC7A11 by inhibiting the FGFR4/GSK-3β pathway, thereby increasing sensitivity to ferroptosis [85]. In HER2-positive breast cancer, overactivation of HER2 upregulates SLC7A11 via the PI3K/AKT/mTOR pathway to increase the antioxidant capacity [85]. The combination of chemotherapeutic drugs and ferroptosis inducers can reverse drug resistance in HER2+ breast cancer [86]. For clinical strategies targeting ferroptosis, existing interventions include GPX4 inhibitors, such as red ginseng polysaccharides, which induce ferroptosis by downregulating GPX4. Their combination with immunotherapy can enhance therapeutic efficacy [87]. Epigenetic regulation is prominent in breast cancer ferroptosis. DNA methylation, microRNAs (miRNAs), and m6A modifications differentially regulate the expression of ferroptosis-related genes [88]. High methylation of the SLC7A11 promoter suppresses its expression and increases cellular resistance to ferroptosis. m6A demethylation activates the SLC7A11/FPN1 axis by inhibiting the FGFR4/GSK-3β/β-catenin pathway, suggesting a novel strategy to overcome resistance to HER2 treatment [85]. The METTL3 inhibitor STM2457 promotes GPX4 degradation by reducing m6A modification, inducing ferroptosis and inhibiting metastasis in TNBC [89]. Noncoding RNAs and ferroptosis have complex relationships in cancer progression. Different ncRNAs can either promote or inhibit ferroptosis in cancer cells. ncRNA-mediated regulation of ferroptosis may represent a new therapeutic direction for treating breast cancer. Long noncoding RNAs (lncRNAs) contribute to improving prognosis and identifying potential therapeutic targets in breast cancer [90]. LncFASA promotes lipid droplet formation and inhibits lipid peroxidation by activating the SLC7A11‒GPX4 axis [91]. The mechanism of tumour suppression involves multiple pathways. LINC00152 confers tamoxifen resistance by inhibiting ferroptosis through BACH1, and sensitivity to endocrine therapy can be restored by silencing LINC00152 [92]. Additionally, the lncRNA H19 regulates chemotherapy resistance in breast cancer via the DNA damage response pathway [93].

### Cuproptosis

Cuproptosis is a recently identified form of copper-dependent PCD that differs from apoptosis, necroptosis, and ferroptosis. Specifically, the accumulated copper ions within cells directly bind to lipoylated proteins in the TCA cycle, leading to their inactivation and subsequent cell death (Fig. 3) [94]. Notably, breast cancer cells exhibit elevated aerobic respiration due to their highly active mitochondrial metabolism, which may potentiate tumour angiogenesis via the cuproptosis pathway, thereby fostering a tumour-promoting microenvironment [95]. Copper homeostasis has a dual nature in that it enhances the capacity of copper ions as cofactors for SOD to scavenge reactive oxygen species [96]. Conversely, excessive Cu⁺ can generate ·OH via the Fenton reaction, inducing oxidative stress and cellular damage [97]. In this context, GSH can remarkably maintain copper homeostasis by neutralising ROS toxicity through reduction reactions [98]. During the cuproptosis process, the key target FDX1 interacts with components of the tumour microenvironment. The mitochondrial protein FDX1 has been validated as a target of the anticancer drug Elesclomol, which induces specific copper-mediated cell death by facilitating copper ion delivery [99]. Notably, the hypoxic microenvironment characteristic of TNBC markedly suppresses FDX1 expression. Under these conditions, lipoic acid synthase sustains TCA cycle function through Fe‒S cluster-mediated acylations [100].

In epigenetics, lncRNAs associated with cuproptosis significantly regulate the proliferation and metastasis of breast cancer cells and potentially predict prognosis and sensitivity to various therapies [101]. The copper transporter SLC31A1 is aberrantly overexpressed in breast cancer cells and is significantly positively correlated with tumour immune infiltration and the expression of immune checkpoint molecules such as PD-L1 [102]. SLC31A1 is significantly correlated with diverse immune cell infiltrations, immune cell biomarkers, and immune checkpoints in breast cancer and is regulated by the LINC01640/miR-204-5p/SLC31A1 axis, which may be central to copper-induced cell death in breast cancer, suggesting that SLC31A1 may serve as a potential target for adjuvant immunotherapy [103].

### Oxeiptosis and disulfidptosis

#### Oxeiptosis

**Fig. 4:**
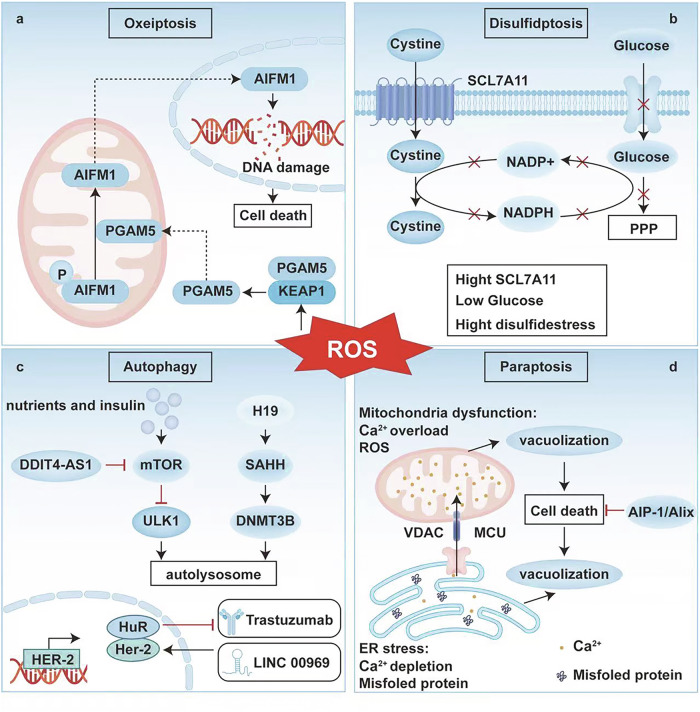
Mechanisms of cellular oxeiptosis, disulfidptosis, autophagy and paraptosis. **a** *Schematic diagram of the oxeiptosis pathway*. After being phosphorylated, AIFM1 in mitochondria is translocated to the nucleus under the action of PGAM5, where it causes DNA damage and ultimately leads to cell death. This process is regulated by KEAP1. **b** *Schematic diagram of the disulfidptosis pathway*. Under glucose-deprivation conditions, the supply of NADPH becomes limited, leading to excessive accumulation of cysteine and other disulfide-containing molecules in SLC7A11-high cells. This ultimately induces a disulfide stress state, which triggers rapid disulfide-dependent cell death. **c** *Schematic diagram of the autophagy pathway*. Nutrients and insulin signals can activate mammalian targets of mTOR. DDIT4-AS1 can inhibit mTOR, thereby activating ULK1 and promoting the formation of autophagosomes. In addition, H19 can affect the autophagy process by regulating SAHH and DNMT3B or through the HER2/HuR/LINC00969/trastuzumab axis. **d** *Schematic diagram of the paraptosis pathway*. Endoplasmic reticulum stress leads to Ca²⁺ depletion and the accumulation of misfolded proteins. Mitochondrial dysfunction causes Ca²⁺ overload and ROS generation, resulting in mitochondrial vacuolisation and ultimately cell death mediated by AIP-1/Alix. MANF: Neurotrophic factor; MAPK: Mitogen-activated protein kinase. AIFM1: Apoptosis-inducing factor mitochondrial 1; P: Phosphorylated; PGAM5: Phosphoglycerate mutase 5; PPP: Pentose phosphate pathway; NADPH: Nicotinamide adenine dinucleotide phosphate; NADP⁺: Nicotinamide adenine dinucleotide phosphate; Ca²⁺: Calcium ion; ROS: Reactive oxygen species. The figure was created with BioRender.com.

Oxeiptosis is a noninflammatory form of cell death initiated by oxidative stress and is sensitive to ROS [104]; this form of cell death is carried out primarily through the KEAP1/PGAM5/AIFM1 signalling pathway, thereby protecting the organism from ROS-induced damage and inflammation [105]. As shown in Fig. 4a, excessive accumulation of ROS triggers KEAP1 activation and enables KEAP1 to interact with PGAM5. This interaction facilitates the dephosphorylation of the intermembrane space protein AIFM1 by PGAM5, which in turn triggers the release and nuclear translocation of AIFM1, ultimately leading to large-scale DNA fragmentation [106]. Alloimperatorin enhances the expression of KEAP1 without altering the expression levels of PGAM5 or AIFM1, thereby inhibiting the proliferation and invasion of breast cancer cells [106].

#### Disulfidptosis

When glucose is depleted, the cellular redox state becomes insufficient. Mesencephalic astrocyte-derived neurotrophic factor (MANF) mitigates protein oxidation and restores phagocytosis mediated by E3 ligase activity, thereby promoting the survival of BC cells under glucose starvation [106]. Cystine transported by SLC7A11 induces the response of actin cytoskeleton proteins to disulfide stress, leading to disulfide-induced apoptosis [107]. Central genes associated with disulfide-related immune checkpoints can predict the prognosis of breast cancer [108]. Breast cancer subtypes can be predicted by lncRNAs involved in disulfide metabolism. Specifically, in the basal subtype, LINC02188 is highly expressed, whereas LINC01488 and GATA3-AS1 exhibit the lowest expression levels [109]. LINC00511 is expressed at the highest level in the Her2 subtype, and GATA3-AS1 is expressed at the highest level in the LumA, LumB, and normal-like subtypes [108]. These findings provide a novel direction for identifying clinical therapeutic targets for breast cancer (Fig. 4b) [109].

### Autophagy

Autophagy eliminates damaged organelles and abnormal proteins via the lysosomal degradation pathway to maintain intracellular homeostasis and suppress genomic instability [110]. Under physiological conditions, moderate levels of autophagy effectively mitigate both endogenous and exogenous inflammatory responses by inhibiting NLRP3 inflammasome activation [111]. Notably, breast cancer cells achieve metabolic adaptation by hijacking the autophagy mechanism: under glucose-deprivation conditions, MANF-mediated mitophagy eliminates dysfunctional mitochondria through the PRKN/Parkin-dependent pathway, maintains the ATP supply and promotes tumour survival [112], suggesting that targeting the MANF‒PRKN interaction can break the drug resistance barrier of breast cancer under metabolic stress.

Autophagy and ferroptosis involve complex metabolic interactions: autophagy-dependent degradation of ferritin leads to the accumulation of free iron and promotes lipid peroxidation through the Fenton reaction. Meanwhile, mitochondrial damage mediated by PCD protein 2 can increase the generation of mitochondrial reactive oxygen species, synergistically inducing autophagy and ferroptosis [113]. Notably, the combination of autophagy inhibitors (such as chloroquine) and ferroptosis inducers (such as erastin) can significantly inhibit breast cancer proliferation, suggesting the potential of synergistic therapy for dual death pathways [114]. Noncoding RNAs exert regulatory functions within the autophagy network [115]. LncRNAs significantly modulate autophagy plasticity via epigenetic and posttranscriptional mechanisms. For example, DDIT4-AS1 promotes protective autophagy by inhibiting mTORC1 complex activity, thereby facilitating the progression of triple-negative breast cancer. Its silencing enhances sensitivity to cisplatin-induced DNA damage [116]. H19 activates autophagy through the H19/SAHH/DNMT3B axis, potentially contributing to tamoxifen resistance in breast cancer (Fig. 4c) [117].

### Paraptosis

Paraptosis represents an atypical form of PCD [118]. The key features that distinguish paraptosis from classical apoptosis include its independence from caspase-9 activation, the absence of typical apoptotic markers such as chromatin condensation or DNA laddering, and cytoplasmic vacuolisation as the predominant morphological feature positively modulated by the mitogen-activated protein kinase (MAPK) signalling pathway [119]. Additionally, paraptosis can be specifically inhibited by the apoptosis inhibitory protein AIP-1/Alix, indicating a distinct regulatory network separate from the apoptotic pathway [120]. The occurrence of paraptosis is closely associated with dysfunction in the ER‒mitochondrial axis [121]. The accumulation of misfolded proteins within the ER lumen leads to osmotic imbalance, resulting in water efflux and expansion of the ER compartment. Persistent ER stress promotes the extensive release of Ca^2+^ into the cytoplasm via the IP3R channel. Ca^2+^ subsequently enters the mitochondrial matrix through the mitochondrial calcium uniporter to cause mitochondrial swelling and membrane potential collapse, ultimately leading to cell death (Fig. 4d) [122, 123]. Research has demonstrated that celastrol induces paraptosis in breast cancer cells via the IP3R-dependent Ca^2+^ signalling pathway, highlighting the critical role of Ca^2+^ homeostasis disruption in this death pattern [124]. Increasing attention has been given to emerging therapeutic strategies based on the paraptosis mechanism [125].

## Difficulties and innovative treatments in breast cancer therapy

### The central role of reactive oxygen species in drug resistance mechanisms

ROS serve as a central regulatory hub in the development of drug resistance phenotypes in breast cancer. The mechanisms promoting drug resistance include the following: first, at the pharmacokinetic level, the overactivation of drug efflux pumps is mediated by ATP-binding cassette transporters (ABC transporters); second, at the molecular biology level, this involves epigenetic modifications of drug targets, activation of intracellular drug-metabolising enzymes, and abnormal activation of epithelial‒mesenchymal transition processes and DNA damage repair pathways regulated by key signalling pathways such as the PI3K/AKT and NF-κB pathways [126]. Notably, clinical observations indicate that endocrine therapy resistance is closely associated with oestrogen-dependent redox signal dysregulation mediated by ROS, suggesting that targeting ROS homeostasis may represent a novel breakthrough for reversing drug resistance [127]. Myeloperoxidase (MPO) generates ROS [128]. In a rigorous clinical trial, the Southwest Oncology Group SWOG-8897 found that the MPO genotype of breast cancer patients undergoing chemotherapy was associated with a lower risk of recurrence, and a high-activity MPO genotype was related to a higher survival rate among women treated with cyclophosphamide [129]. Memo is a copper-dependent redox enzyme that facilitates the production of ROS [130]. A greater than 40% increase in Memo expression has been linked to adverse clinical parameters in primary breast cancer and serves as an independent prognostic marker for early distant metastasis [121].

### Nanodelivery systems enhancing photodynamic therapy

Photodynamic therapy (PDT) relies on ROS generation through photosensitizers under specific wavelength excitation. PDT can kill tumour cells but is limited by the low bioavailability of phototherapeutic agents. Nanoparticles have emerged as ideal carriers for optimising PDT due to their precise targeting and high drug loading efficiency [131]. Nanoparticles have become powerful and multifunctional tools for inducing ROS to achieve effective cancer therapies [132]. Preclinical studies have demonstrated that the sequential application of photodynamic therapy and radiotherapy significantly improves therapeutic outcomes for triple-negative breast cancer, highlighting the potential of combined therapies [133]. In a clinical trial, PDT was applied to 7 patients with breast cancer recurrence on the chest wall to relieve chest wall pain [134]. The results showed that the overall response rate was 91%, with a complete response rate of 73% and a partial response rate of 18%, indicating that this treatment plan has significant clinical efficacy [134].

### Multiple catalytic effects of nanozymes

Nanozymes, characterised by their biomimetic catalytic activity, high stability, and programmable nature, offer a novel strategy to address the limitations of traditional enzyme preparations [135]. Specifically, nanozymes can induce immunogenic cell death through various mechanisms, such as alloy nanoparticles like Cu-Ag NPs [136] or FeCu-DA [137], as well as nanozyme platforms [138]. Notably, the PtMnIr nanozyme exhibits multiple enzymatic activities, integrating OXD, CAT, SOD, POD, and GPX functionalities, thereby promoting ferroptosis and apoptosis in tumour cells while inhibiting tumour metastasis by establishing an “endogenous GSH consumption–ROS cyclic generation” mechanism [139]. In addition, several studies have demonstrated the vital role of nanozymes in the research and treatment of breast cancer [140]. Currently, the catalytic activity of nanozymes is utilised to locally amplify ROS stress in breast cancer, and targeted therapy is achieved through carrier design [141]. The specific nanozymes and their corresponding mechanisms are illustrated in Table 2.

**Table 2:** Some of the reported studies using Nanozymes in breast cancer.

Nanozymes	Mechanism	Type of breast cancer	Reference
CuFeSe2	Conversion of endogenous H2O2 to hydroxyl radical (•OH)	TNBC	[144]
FZSHC	Consumption of glutathione affected the calcium metabolism of tumour cells	BC	[145]
ES@Cu (II)-MOF	Reduce the pH of lysosomes catalyses	BC	[147]
MnN5 SA/CNF	Combine SCT and induce apoptosis of cells through mitochondria	BC	[148]
PtNEs	Enhance photodynamic therapy	BC	[148]
FeSHS	Disrupt FSP1/CoQ10/NAD(P)H axis	BC	[150]
Au@Pd nanozyme	Generates a large amount of ROS, leading to DNA damage and cell death	BC	[151]

### Tumour microenvironment (TME)-targeted nanotherapy

Reprogramming the TME is a critical direction in nanotherapy, with strategies including immune cell regulation and metabolic intervention. Stimulatory nanoparticles can reprogramme antigen-presenting cells and enhance T-cell activation, as demonstrated in the 4T1 model [142]. Paclitaxel nanoformulations induce macrophage polarisation to the M1 phenotype via the TLR4/cGAS-STING pathway, thereby reversing the immunosuppressive microenvironment [143–145]. Compared with standard paclitaxel, biologically interactive albumin-bound paclitaxel in solvent-free nanoparticles demonstrated greater efficacy and good safety [146]. The PFTT@CM nanosystem developed by Pan et al. enhances PDT efficacy by depleting GSH and inducing ferroptosis [147]. Additionally, ILA@Lip creates a highly hypoxic tumour microenvironment, reduces angiogenesis and inhibits tumour metastasis [148]. These advancements underscore that nanoparticles can precisely regulate TME, which is of great significance for personalised treatment [149].

### Sonodynamic-nano synergistic therapy

The fundamental principle of sonodynamic therapy (SDT) involves utilising ultrasound to stimulate sonosensitizers to activate cavitation activity and promote the generation of ROS, followed by inducing the apoptosis of harmful cells such as tumour cells and bacteria [150]. SDT employs ultrasound to activate sonosensitizers for ROS production, with tissue penetration superior to that of photodynamic therapy. However, its efficacy is constrained by the heterogeneity of the tumour microenvironment [151]. Consequently, developing new sonosensitizers with enhanced sonosensitizing activity remains a significant challenge for SDT technology. The therapeutic efficacy against TNBC can be improved using tumour microenvironment-responsive nanoparticles and epigenetic reprogramming [152]. For instance, constructed HER3-modulated nanobioparticles can cross the blood‒brain barrier and target HER3 [153]. Given the hypoxic nature of the tumour microenvironment, clinical application of SDT remains challenging. Wu et al. proposed a synergistic strategy that combines oxygen-enhanced sonodynamic therapy with ferroptosis via engineered exosomes, demonstrating higher efficacy in anticancer treatment [154]. Moreover, the combination of sonodynamic therapy with localised chemotherapy can mitigate the adverse effects of chemotherapeutic agents on healthy tissue [155]. Currently, clinical trials investigating sonodynamic therapy are limited to brain tumours [156], and research on its application in breast cancer remains to be further explored.

## Discussion

This review systematically elucidates the pivotal role of ROS in regulating multiple PCD pathways and highlights the potential therapeutic value of targeting the ROS–PCD network. ROS serves as both a pro-cancer signalling molecule and an executor of cell death, making it a critical node linking tumour metabolism, epigenetic regulation, and microenvironmental modulation. A comprehensive understanding of the balance between oxidants and antioxidants in breast cancer is essential for developing effective treatment strategies. The various PCD pathways in breast cancer are interconnected through shared molecular nodes, forming a complex regulatory network. Beyond apoptosis, other forms of cell death crosstalk also exist in breast cancer. The same stimulus can induce distinct forms of cell death depending on the specific situation. For example, lapatinib induces ferroptosis via iron-dependent ROS generation in the early stages but promotes protective autophagy to facilitate drug resistance in the later stages [157]. High concentrations of copper ions can activate autophagy through lysosome-dependent mechanisms [158]. Additionally, the long noncoding RNA LINC00618 orchestrates cell fate through dual mechanisms; LINC00618 inhibits cystine uptake by downregulating SLC7A11 to promote ferroptosis and simultaneously induces apoptosis by upregulating BCL-2-BAX expression and activating caspase-3 [52]. This multitarget regulatory pattern underscores the central role of epigenetic modifications in maintaining the dynamic equilibrium of PCD pathways. Therefore, investigating epigenetic regulation across different cell death states to unravel the progression of breast cancer is crucial. Based on current advancements in oxidative stress research in breast cancer, we propose several innovative therapeutic approaches targeting the ROS–PCD network for future development: (1) Oxidative stress-directed small-molecule drugs: Natural compounds such as curcumin can induce apoptosis by triggering mitochondrial ROS bursts and DNA damage while enhancing ferroptosis sensitivity through GPX4 inhibition. However, their low bioavailability and off-target effects limit their clinical application. Nanodelivery systems are required to optimise the targeting efficacy. Compared with free curcumin, curcumin encapsulated in NPs demonstrates enhanced anti-proliferative effects [159], offering a promising strategy for breast cancer treatment. (2) Epigenetic intervention: Targeting epigenetic modifications can reshape the expression profiles of PCD-related genes. DNA methyltransferase activates long noncoding RNA PHACTR2-AS1 to suppress PH20 expression, thereby controlling the growth and metastasis regulatory axis of epigenetic regulation across different cell death states [160]. The DNMT1-PAS1-PH20 axis represents a potential therapeutic target for breast cancer [160]. (3) Nanoimmune combination therapy: Multifunctional nanoplatforms can overcome TME barriers by integrating catalytic activity and immune modulatory functions. For instance, cascade multienzyme nanoparticles (e.g., PtMnIr) can deplete GSH, continuously generate ROS, and release damage-associated molecular patterns (DAMPs) to promote dendritic cell maturation and cytotoxic T lymphocyte infiltration [161]. The combination of these platforms with immune checkpoint inhibitors (e.g., α-PD-L1) significantly suppresses primary tumours and distant metastases, shedding new light on immunotherapy in “cold tumours” [161]. A search for “breast cancer” and “nanoparticles” in the ClinicalTrials.gov database reveals the number of clinical trials focusing on nanotechnology-based breast cancer treatments. As of 2025, a total of 81 clinical trials have been initiated in this area, with four of them having been withdrawn. In recent years, the number of clinical studies investigating nanoparticle-based therapies for breast cancer has steadily increased (ClinicalTrials.gov, 2025). However, complex interactions and safety concerns exist between nanoparticles and sonodynamic therapy. Consequently, no clinical trials have been initiated in this area to date, and significant challenges remain in the clinical translation of nanomedicines. In summary, despite the challenges posed by tumour heterogeneity, diverse microenvironmental adaptability, and safety concerns, targeted therapies aimed at ROS and related PCD pathways offer new perspectives for precision medicine in breast cancer. Future efforts should focus on full-chain innovation from mechanistic exploration to therapeutic breakthroughs through advanced drug design and closed-loop preclinical-clinical translation.

## Conclusion

Breast cancer, the leading cause of cancer-related deaths among females worldwide, faces significant challenges due to its high heterogeneity and drug resistance. This article systematically examines the dual role of ROS in the initiation, progression, and treatment resistance of breast cancer and explores innovative therapeutic strategies targeting ROS-related PCD. In conclusion, targeting ROS and associated PCD pathways offers a promising breakthrough for the treatment of breast cancer. However, clinical success hinges on the deep integration of interdisciplinary technological innovation within the framework of precision medicine. Future research should focus on establishing a closed loop from mechanism to technology and clinical translation, ultimately achieving substantial improvements in the survival of patients. Although there remain several issues to be carefully considered, the enormous potential of these nanoparticles deserves further investigation. We hope this review can provide comprehensive insights to facilitate the therapeutic progress of breast cancer.
